# Understanding the foundations of the structural similarities between marketed drugs and endogenous human metabolites

**DOI:** 10.3389/fphar.2015.00105

**Published:** 2015-05-13

**Authors:** Steve O'Hagan, Douglas B. Kell

**Affiliations:** ^1^School of Chemistry, The University of ManchesterManchester, UK; ^2^The Manchester Institute of Biotechnology, The University of ManchesterManchester, UK

**Keywords:** drug transporters, cheminformatics, endogenites, metabolomics, encodings

## Abstract

**Background:** A recent comparison showed the extensive similarities between the structural properties of metabolites in the reconstructed human metabolic network (“endogenites”) and those of successful, marketed drugs (“drugs”).

**Results:** Clustering indicated the related but differential population of chemical space by endogenites and drugs. Differences between the drug-endogenite similarities resulting from various encodings and judged by Tanimoto similarity could be related simply to the fraction of the bitstrings set to 1. By extracting drug/endogenite substructures, we develop a novel family of fingerprints, the Drug Endogenite Substructure (DES) encodings, based on the ranked frequency of the various substructures. These provide a natural assessment of drug-endogenite likeness, and may be used as descriptors with which to derive quantitative structure-activity relationships (QSARs).

**Conclusions:** “Drug-endogenite likeness” seems to have utility, and leads to a simple, novel and interpretable substructure-based molecular encoding for cheminformatics.

## Introduction

In a recent study (O'Hagan et al., 2015), motivated by the recognition that drugs do, and probably have to, hitchhike on metabolite transporters in order to get into cells (Dobson and Kell, 2008; Dobson et al., 2009a,b; Giacomini et al., 2010; Kell et al., 2011, 2013, 2015; Kell, 2013, 2015; Kell and Goodacre, 2014; Kell and Oliver, 2014), we have used the recent availability of a curated reconstruction of the human metabolic network, Recon2 (Swainston et al., 2013; Thiele et al., 2013), to ask the question as to how similar in structural terms marketed drugs are to the molecules (hereafter “endogenites”) involved in endogenous human metabolism. While the results depended quite considerably on the exact 2D descriptor used to encode the structures, it was noted that for the commonly used MACCS166 descriptor (Durant et al., 2002; Todeschini and Consonni, 2009) in the implementation described (and see http://www.dalkescientific.com/writings/diary/archive/2014/10/17/maccs_key_44.html), there was at least one endogenite with a Tanimoto similarity (TS) exceeding 0.5 for more than 90% of marketed drugs. As noted in those references (Durant et al., 2002; Todeschini and Consonni, 2009), the MACCS166 descriptor consists of a string of 166 binary elements representing the presence or absence of 166 (slightly arbitrary and not necessarily druglike) features. We note that not all the MACCS keys represent substructures, some are rather simple, e.g., “has one or more element [x] atoms.” Most of the cheminformatic tool kits (e.g., RDkit, CDKit) are implemented using SMARTS queries; these can only approximate the original MDL MACCS keys. In some cases the intended behavior of the key (query) was ambiguous, in other cases, a SMARTS query is unable to replicate the original MDL query as intended. Nevertheless, the various toolkit MACCS fingerprints are claimed to be sufficiently close to the original MDL versions. The 166 subset were based on the MDL MACCS key that were made public. The RDKit implementation is described at http://rdkit.org/Python_Docs/rdkit.Chem.MACCSkeys-pysrc.html.

It was concluded that while this “does not mean, of course, that a molecule obeying the rule is likely to become a marketed drug for humans, it does mean that a molecule that fails to obey the rule is statistically most unlikely to do so” (O'Hagan et al., 2015), implying that the degree of endogenite-likeness could indeed be a useful chemical filter in drug discovery programmes. Others too have noted the general “natural metabolite-likeness” of drugs (e.g., Feher and Schmidt, 2003; Karakoc et al., 2006; Gupta and Aires-De-Sousa, 2007; Dobson et al., 2009b; Khanna and Ranganathan, 2009, 2011; Peironcely et al., 2011; Zhang et al., 2011; Chen et al., 2012; Walters, 2012; Hamdalla et al., 2013; Manallack et al., 2013), often using supervised methods of machine learning, though in our own work (O'Hagan et al., 2015), especially to avoid the dangers of overtraining (Broadhurst and Kell, 2006), we purposely confined ourselves to using unsupervised methods only. We also noted (O'Hagan et al., 2015) that a rather smaller fraction of molecules in typical drug discovery libraries obeyed the rule.

**Figure 1:**
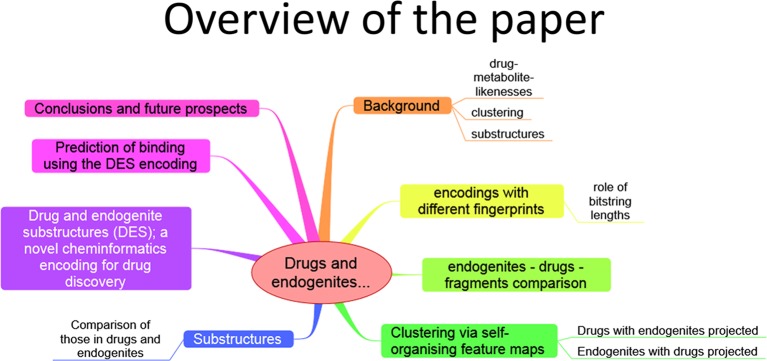
**A “mind map” of the manuscript**.

Partly for reasons of space, however, the previous study (O'Hagan et al., 2015) left a considerable number of questions rather open. These included, for instance, which fingerprint method might be most “suitable” (and whether “better” ones existed), whether similarity measures should be based on a suitable fusion of the results from using different fingerprints (e.g., Ginn et al., 2000; Hert et al., 2004; Whittle et al., 2006; Gardiner et al., 2009; Chen et al., 2010; Medina-Franco et al., 2011; Willett, 2013a,b), which substructures were most important in determining endogenite-likeness, which parts of metabolite space were most fully populated by drugs, whether results differed markedly if we used other clustering methods, and so on. The purpose of the present paper is to develop and provide some of these analyses. It is concluded that drugs are indeed like metabolites when viewed in a variety of orthogonal ways, and that the substructures found within endogenites and marketed drugs provide a novel and useful means of encoding chemical structures in a simple and easy-to-understand manner. Figure 1 gives an overview of the paper in the form of a “mind map” (Buzan, 2002).

## Materials and methods

### Molecular data

We used the same molecules for marketed drugs as before (O'Hagan et al., 2015); they were provided in their entirety as Supplementary files to that paper (O'Hagan et al., 2015) and are not reproduced here. The number of endogenites was lowered to 1057 to remove wildcards in lipids with variable chain lengths, since for some purposes we were here specifically interested in molecular weights, but the endogenites were otherwise identical too. Data for Maybridge fragments and Chembridge molecules were downloaded from their respective websites, and other data were downloaded as indicated in the text.

### Software

We used the KNIME environment (Berthold et al., 2008; Mazanetz et al., 2012; Meinl et al., 2012) throughout, along with a variety of its cheminformatics toolkits such as CDK (Beisken et al., 2013) and RDKIT (Riniker et al., 2013). Details were as given previously (O'Hagan et al., 2015) (and note that the MACCS fingerprints there were not hashed; a correction has been appended at the journal). Quite a few of the nodes used R code, written by O'Hagan and incorporated into the “R Snippet” KNIME node, with substructure counting via the RDKit Substructure Counter node.

## Results and discussion

### Fingerprints

Even (as in O'Hagan et al., 2015) using just 2D fingerprints, the apparent closeness of drug and endogenite molecules to each other (as judged by their Tanimoto similarity coefficients) was differentially “rugged” (the hierarchical clustering showed many more small clusters for drugs than for metabolites), and could differ quite substantially depending on which fingerprint was used (see also e.g., Eckert and Bajorath, 2007; Leach and Gillet, 2007; Faulon and Bender, 2010; Koutsoukas et al., 2014; Maggiora et al., 2014; Medina-Franco and Maggiora, 2014). To explore this further, we decided to compare the drug and metabolite spaces, alone and with each other, using a modification of the approach. Because, of course, the nearest metabolite to itself has a TS of 1, we decided to proceed as follows:

1. For each querying molecule (whether a drug or an endogenite) rank the queried molecules (whether drug or endogenite) and determine the TS of the 90th percentile of closeness.
2. Do this for each fingerprint encoding.
3. For each query molecule and each queried molecule, find the maximum value of the TS among the eight fingerprints tested.
4. Plot the TS of the 90th percentile of the queried molecule against the fraction of the querying molecules tested.

**Figure 2:**
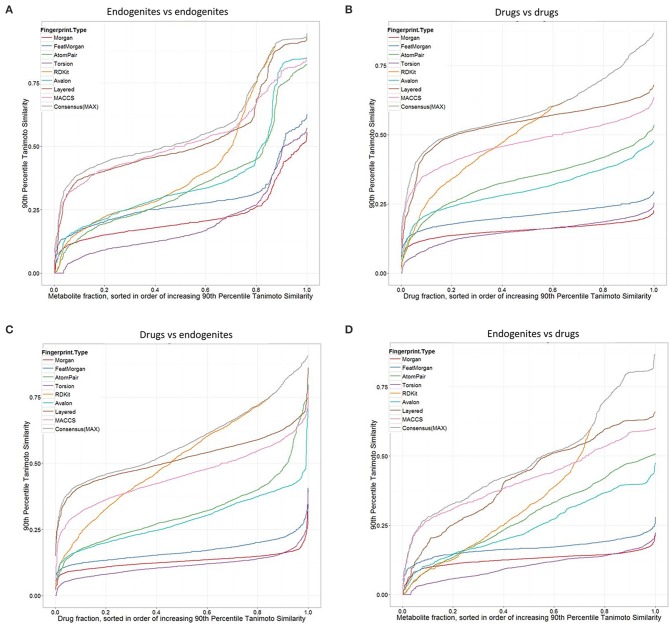
**Tanimoto similarities ranked according to the query target closest at the 90th percentile, for different fingerprint types. (A)** Endogenites vs endogenites. **(B)** Drugs vs drugs. **(C)** Drugs (query) vs. endogenites. **(D)** Endogenites (query) vs. drugs. Consensus (MAX) refers to the “maximum” TS (Gardiner et al., 2009) among the different fingerprints when only the nearest metabolite is returned.

Considering first the endogenites (as compared to each other), we see (Figure 2A) that the RDKIT encoding shows the greatest similarities for metabolites that are *ranked* as being the most similar, but that MACCS and Layered encoding preserve the greater appearances of similarity as the overall similarities decrease. Using these encodings, 40–50% of molecules still had molecules whose TS at the 90 th percentile was 0.5 or above. By contrast (Figure 2B), these fractions were uniformly lower for drugs vs drugs, consistent with the rather spikier or “patchy” population of the normalized chemical space relative to that of endogenites (many of which, especially CoA and steroid/sterol derivatives, share many structural similarities) (O'Hagan et al., 2015). The drug-endogenite comparison (Figure 2C, with the drugs being the query molecules) gives data broadly similar to those shown in Figure 2A of O'Hagan et al. (2015) where closeness to only the very nearest metabolite was plotted, consistent with a view that a querying drug is more commonly close in structural terms not just to a single endogenite but to many such that occupy that part of endogenite space. Figure 2 also shows the data for the “maximum” TS (Gardiner et al., 2009) among the different fingerprints when only the nearest metabolite is returned. Finally, the complementary endogenite-drug comparison, with the endogenite being the query molecule, shows similar but complementary behavior (Figure 2D). One conclusion, given the fact that more than 90% of marketed drugs are seen to be similar to at least some metabolites, and that one might therefore wish to use this as a filter in the analysis of candidate drug libraries, is that for these kinds of comparisons the MACCS, RDKit, Layered or “maximum” fingerprint choice is most likely to return such a result.

**Figure 3:**
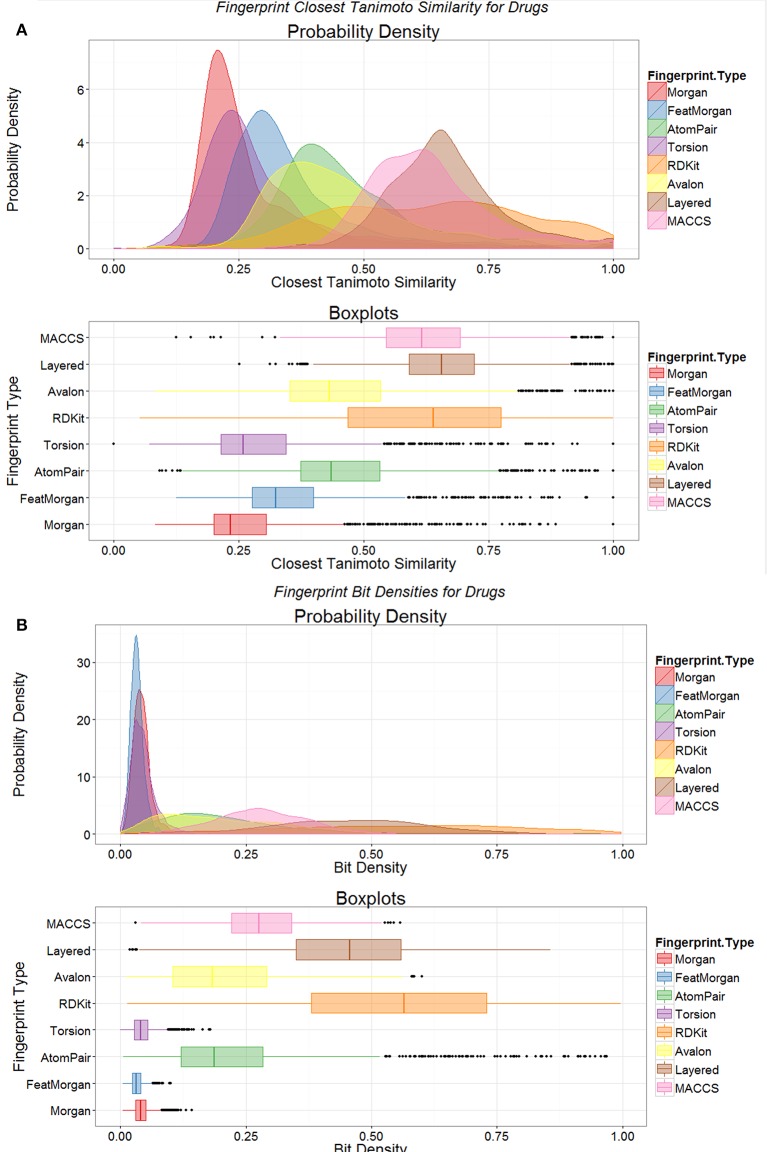
**Distributions of fingerprint properties of drugs. (A)** Distributions of Tanimoto similarities between drugs and endogenites using eight different encodings, shown as probability densities (upper) and boxplots (lower); the boxplots show the median and interquartile range, with the end of the “whiskers” being at 1.5 times the interquartile range, and with extreme examples being given as dots. **(B)** Variation of the probability density of the number of bits set to 1 in the various encodings in **(A)**.

Another way of looking at such data is to compare the *distributions* of the nearest Tanimoto similarities between marketed drugs and metabolites for the different encodings (Figure 3A). It is clear from such a plot (Figure 3A) that not only is the closeness of the “nearest” metabolite different for the different encodings but that the encodings cover metabolite space differentially. At least for the Morgan and Feat Morgan encodings, that resemble ECFP and FCFP (Landrum et al., 2011), this can be ascribed in part to the much smaller number of bits in the encoding that have the value 1 (Figure 3B), since the value for the TS is partly a function of this (Flower, 1998; Godden et al., 2000; Holliday et al., 2002, 2003; Wang et al., 2007; Al Khalifa et al., 2009). [In a similar vein, we also looked at the use of a strategy that doubles the length of the bitstring encoding by adding its complement (Knuth, 1986), such that 50% of the bits are 1 and 50% 0. This was not beneficial, as the high density of zeroes in the original merely doubled the number of similar bits (data not shown).]

**Figure 4:**
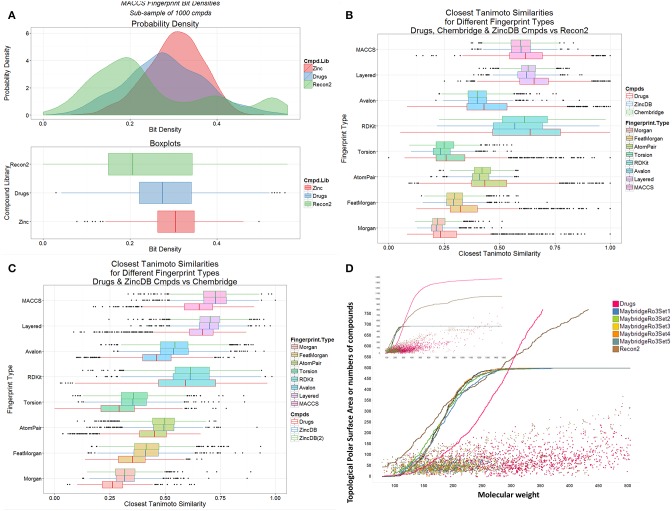
**Differences between marketed drugs, Recon2 and library compounds. (A)** Variation of bit density for the three classes of compound (based on sampling 1000 of each from the three classes). **(B)** Variation of Tanimoto similarity to Recon2 for eight encodings of marketed drugs and library compounds (from Chembridge and from the ZINC database). In each case drugs are more similar to metabolites than are library compounds. **(C)** Variation of Tanimoto similarity of Chembridge library compounds to two subsets [ZINCDB and ZINCDB(2)] of ZINC database compounds and to marketed drugs. In each case library compounds are more similar to each other than to marketed drugs. **(D)** Topological polar surface area and molecular weight distributions of drugs, Recon2 compounds and five “rule-of-3”-compliant (Congreve et al., 2003) libraries of 500 fragments each that are sold for drug screening purposes. The inset is scaled to show all marketed drugs.

We also observed previously that the distribution of metabolite- (endogenite-) likenesses differed significantly between marketed drugs and (many of) the kinds of molecules typically found in drug discovery libraries. A convenient way of encoding these is simply to look at the distribution of bitstring densities (of 1 s) for the appropriate encoding between the molecules (Flower, 1998). Thus, Figure 4A shows that these differ very significantly for random samples taken from Recon2, from marketed drugs, and from the ZINC (Irwin et al., 2012) databases, with drug candidates typically being less like metabolites than are drugs (see also Chen et al., 2012; Walters, 2012), regardless of the database used (Figures 4B,C). The distributions of topological polar surface area (TPSA) and molecular weight (see Abad-Zapatero et al., 2010, 2014) are shown (Figure 4D) for endogenites (Recon2), for marketed drugs, and for 5 libraries of small molecule “fragments” (Maybridge “Ro3”-compatible, Congreve et al., 2003, libraries). For a given molecular weight, endogenites are typically significantly more polar than are marketed drugs or fragments, especially for lower molecular weights. Thus, when compounds are ranked by molecular weight (MW), the median MW for drugs, endogenites and fragments are 335, 291, and 179–185 (depending on the library). For these molecules the TPSA values are 69, 124, and 30–69Å^2^. A noteworthy point (see also Gopal and Dick, 2014), however, is that fully one quarter of marketed drugs are not in fact larger than typical fragments (Figure 4D); indeed when ranked by increasing molecular mass, the 500th marketed drug (of 1383) has a MW of just 297.

We also looked to see whether metabolites that were known substrates (from the Recon2 map) for known transporters (see also Sahoo et al., 2014; Kell et al., 2015) exhibited any greater likelihood to be those with the nearest TS to the query drug; no significant evidence for or against this was found (data not shown), and of course they may be, and may need to be, endogenite-like at their targets too.

## Clustering using self-organizing maps

**Figure 5:**
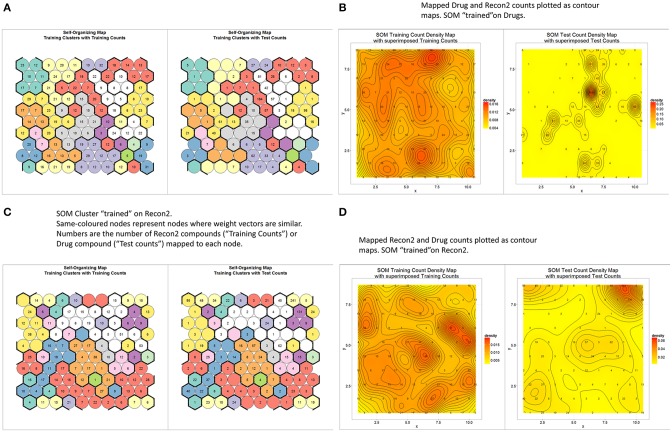
**Relationships between endogenite and marketed drug spaces as judged by self-organizing feature maps trained on marketed drugs as encoded with the MACSS encoding. (A)** A self-organizing map with 100 nodes and 10 clusters, trained to convergence (3000 iterations) (left), along with a projection of endogenites onto the trained network (right). **(B)** The data in A replotted as a contour plot. **(C)** A plot as in **(A)** but the network was trained using the Recon2 endogenite data. **(D)** Contour plot of the data in **(C)**.

Teuvo Kohonen's Self Organizing (Feature) Map (Kohonen, 1989, 2000; Oja and Kaski, 1998) is a well-known unsupervised learning method of clustering data according to a measure of their similarity. It was therefore of interest to see how “drug” and “endogenite” spaces were organized when represented as such a map. To this end, we used the MACCS encoding for marketed drugs, with 10 × 10 nodes and 10 clusters (numbers chosen to give a reasonable but not excessive degree of clustering, given the number of drugs). Figure 5A (left side) shows the distribution of the different numbers of drugs as clustered (by color, based on the similarity of their weight vectors) into the different nodes (circles), while the right hand side of the same figure represents a projection of Recon2 metabolites as projected onto the trained network. The number of circles for each cluster varies quite significantly, from 2 to 15, while the heterogeneous distribution of metabolites shows clearly that some parts of drug space are much less close to multiple metabolites than are others (e.g., the “orange”- and “lemon”-colored clusters). This is especially obvious when the data are displayed as a contour map (Figure 5B). In the converse approach, we trained a self-organizing map (SOM) on Recon2; in this case (Figure 5C) the number of nodes per cluster varied from 1 to 21, showing again that metabolite space has some significantly larger clusters than does drug space, while the projection of drugs onto metabolite space (Figure 5D) shows a highly significant clustering into a particular area of metabolite space, consistent with the finding that there was a significant preference for some metabolites (O'Hagan et al., 2015).

## Substructural basis for drug-endogenite likenesses

**Figure 6:**
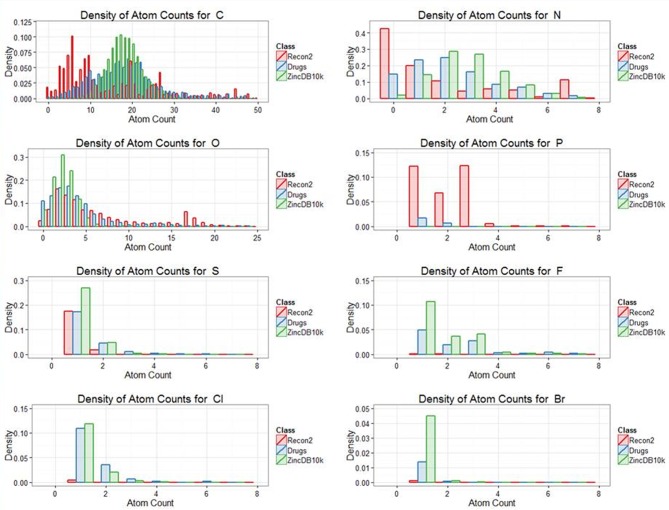
**Distribution of the frequency of appearance different atoms between the classes endogenites, marketed drugs and of 10,000 randomly chosen molecules from the ZINC database**. To maximize visibility, numbers are not plotted if off the ordinate scale.

Our previous analyses of drug-endogenite likenesses looked at the molecules “as a whole.” However, it is obvious that some substructures may be more common in endogenites than in marketed drugs and vice versa, a simple example being the recognition that human endogenites do not contain halogen atoms while various drugs do (e.g., of the 1381 marketed drugs, 148 of them contain at least one fluorine atom). Thus, Figure 6 shows the distribution of atom types for the three classes drugs, endogenites, and library compounds.

Starting arguably with (Bemis and Murcko, 1996, 1999), a number of papers have analyzed the frequency of occurrence in FDA-approved, marketed drugs of various substructures, including heterocycles (Vitaku et al., 2014), rings (Aldeghi et al., 2014; Taylor et al., 2014), the chronological (and relatively recent) appearance of S and F in drugs (Ilardi et al., 2014), and even metallodrugs (Mjos and Orvig, 2014). Papers also exist in which fingerprinting methods have been used to *distinguish* drugs from metabolites (e.g., Khanna and Ranganathan, 2009, 2011; Peironcely et al., 2011; Walters, 2012; Hamdalla et al., 2013). However, while Chen et al. (2012) did note that human metabolites and natural products tended to have fewer terminal rings than do marketed drugs, no one has compared the substructures found in marketed drugs with those found in the human endogenites represented in Recon2, which is what we now do here.

**Figure 7:**
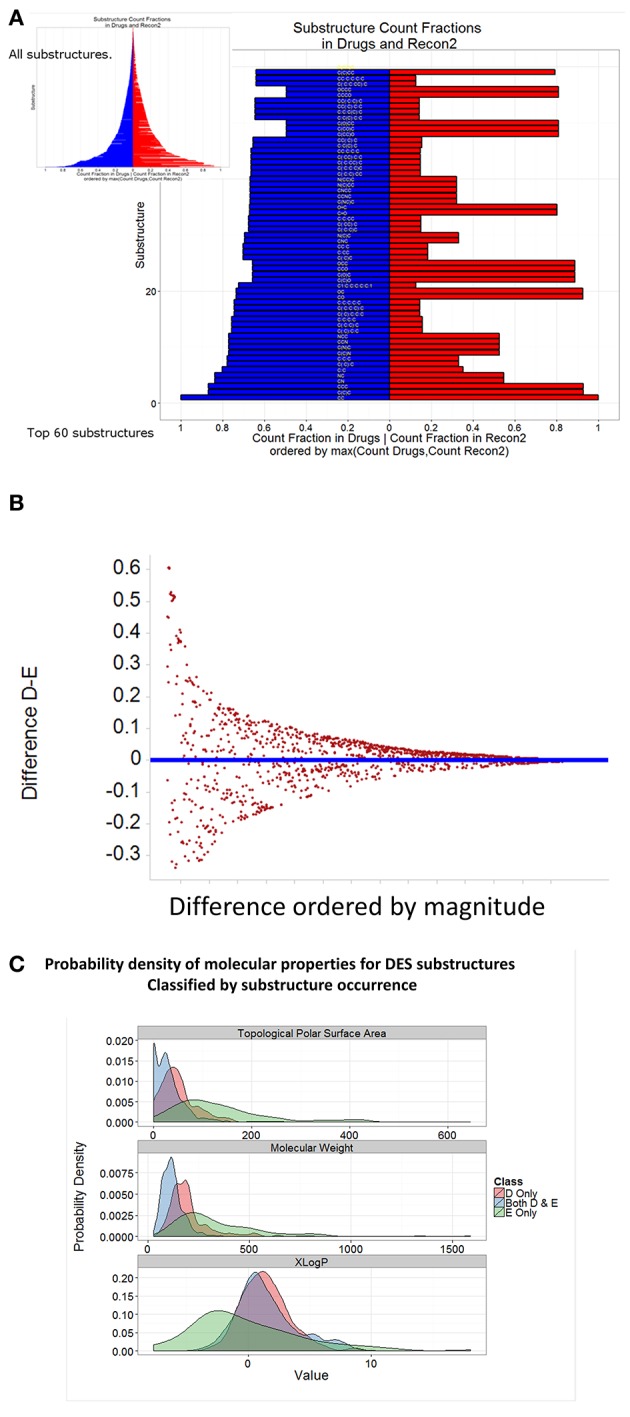
**Frequency of representation of different substructures in endogenites and marketed drugs**. Self-organizing maps were run as in Figure 5 for 10 separate occasions. For each SOM node, using the MCS (maximum common scaffold) analyser from Indigo within KNIME, we extracted all substructures for each SOM node; this was performed 10 times, and duplicates removed. **(A)** Substructures were ranked according to their frequency of appearance in either drugs or endogenites, normalized to the total number of either. **(B)** Difference plot of the data in **(A)**. **(C)** Distribution of three properties of drug and endogenite substructures.

Using the Indigo substructure analyser in KNIME, we extracted relevant substructures from both endogenites and marketed drugs, and ranked them according to the normalized frequency of their appearances. The top 60 substructures in each clade are shown in Figure 7, while all are illustrated diagrammatically in the inset to Figure 7A, with the full Table of data being supplied as Supplementary Information. It is clear from Figures 7A,B that while there are indeed some clear similarities between drugs (blue) and endogenites (red) (Figure 7A), with a greater frequency of more substructures in drugs (Figure 7B), there are also some substantial differences (Figure 7C) in the frequency of various substructures between endogenites and present marketed drugs (those substructures that occur frequently in drugs are sometimes referred to as “privileged,” Tounge and Reynolds, 2004; Costantino and Barlocco, 2006; Schnur et al., 2006). It is probably also worth noting that in some sense substructures may be related to the fragments that have proved so useful in drug screening (e.g., Hall et al., 2014), and that proposals exist that one might concentrate on those that are metabolite-like (Davies et al., 2009) or natural-product-like (Over et al., 2013).

## Use of drug/endogenite substructure presence as an encoding strategy

While some encodings, such as MACCS (Durant et al., 2002), use the presence or absence of particular substructures as the basis for their binary scoring, the substructures so chosen are somewhat arbitrary (or at least not necessarily based on any knowledge of the structures of marketed drugs nor endogenites). Armed with the substructures of Figures 7A–C (Supplementary Information) we used each of the substructures found (whether in endogenites, drugs or both) as a 1419-bit presence/absence encoding, on the basis that these substructures ought at least to form the basis of useful drug molecules in the future, as they must include or contribute to the concept of “drug-likeness” (Muegge, 2003; Lipinski, 2004; Oprea et al., 2007; Abad-Zapatero et al., 2010, 2014; Camp et al., 2012; Garcia-Sosa et al., 2012; Yusof and Segall, 2013), not least since approved drugs occupy only a rather particular subset of the chemical Universe (Ruddigkeit et al., 2012, 2013). We refer to this encoding as the Drug-Endogenite-Substructure (DES) encoding.

**Figure 8:**
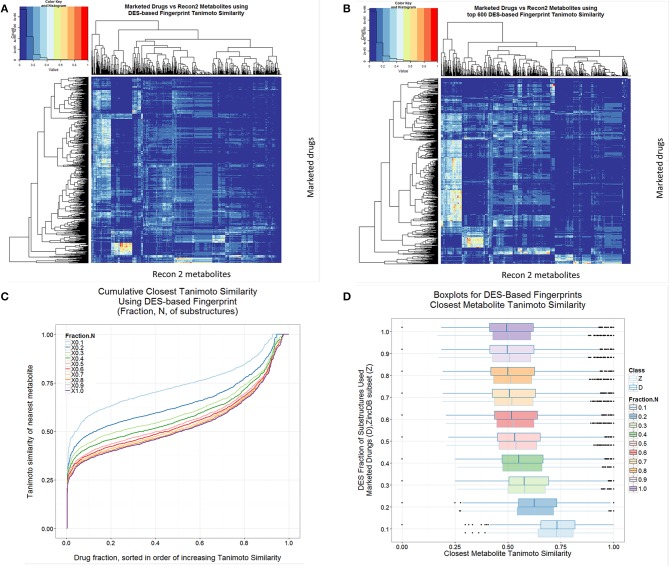
**Drug-endogenite similarities as judged using the DES encoding. (A)** Heat map of endogenites vs. drugs with full DES encoding. **(B)** As A but with the most frequent 600 substructures (^1^DES_600_). **(C)** Cumulative plot of endogenite-likeness using DES encodings based on the fraction of total substructures ordered (from right to left) from the most frequent to least frequent. **(D)** Boxplots of nearest Tanimoto similarities of drugs to endogenites or to ZINC database subsets as the fraction of the DES encoding was varied.

Given its origins and basis, the DES encoding is necessarily likely to indicate more clearly than many encodings the drug-metabolite similarities, and such data are given in Figure 8, both for the full set of substructures so extracted (Figure 8A) and for truncated versions decreased as per the ranking order in the full Supplementary Information (Figures 8B–D). In this case, it is clear that there are advantages in not being too comprehensive, and that using the DES encoding with the top 10% of drug-endogenite substructures results in a drug-endogenite similarity even greater than that found previously [1] using the MACCS encoding; this again would seem to reflect the fraction of bits set to 1 in the bitstring that results from the encoding. This is also true for molecules taken at random from the ZINC database (Figure 8D). The KNIME element that calculates the bitstring from the molecular structure encoded in SMARTS strings was mainly written in R, and is provided as Supplementary File 2 (Scaffold2DES-Fingerprint.7z).

Given the supplementary information it is possible to cut substructures from both the most and least frequently found substructures in the list. We suggest that these encodings might also be useful for various purposes, and might usefully be referred to as ^X^DES_Y_ where X and Y are numbers referring to the first and last of the substructures used. [We note that one might also use something like an evolutionary algorithm for subset selection (e.g., Broadhurst et al., 1997) and other kinds of optimization (Kell and Lurie-Luke, 2015), but as noted above we have chosen to avoid supervised methods for these purposes here.]

**Figure 9:**
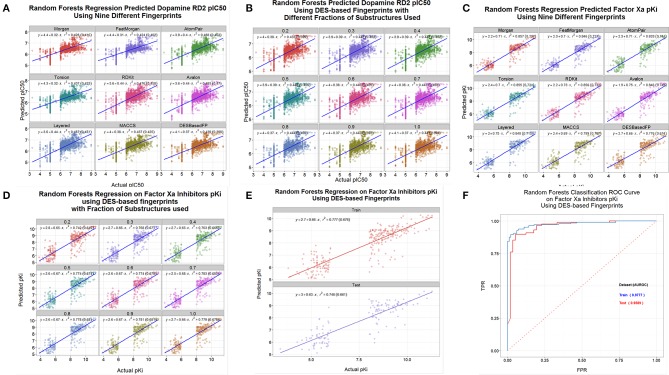
**QSAR and classifier analyses of drug binding using various encodings of drug structures. (A)** A random forest model was learned using the data for drug binding to the dopamine D2 receptor at http://www.bindingdb.org/bind/ByMonomersTargets.jsp?nBindingData=9349&submit=Search. The out-of-bag predictions were made after 2000 trees were added. **(B)** Same as **(A)** save that we used only the fractions of the DES encodings indicated. **(C)** Same as **(A)** save that the data were for factor Xa inhibition (Fontaine et al., 2005) using data downloaded from http://www.cheminformatics.org/datasets/. **(B)** Same as **(C)** save that we used only the fractions of the DES encodings indicated. **(E)** Same as **(C)** save that data were split into training (out of bag predictions) and test sets as per the data at http://www.cheminformatics.org/datasets/. **(F)** Classification of data (using a Receiver Operator Characteristic curve) from **(C)** to **(D)** based on whether the molecule was a “good” or “poor” inhibitor.

A common use of these kinds of encodings is in the calculation of quantitative structure-activity relationships (Geldenhuys et al., 2006; Tropsha, 2010; Stålring et al., 2011; Warr, 2011; Ruusmann et al., 2014). We assessed the ability of the DES and other encodings to predict the binding of various drugs to three candidate targets, using data taken from the internet. Thus, Figure 9A shows the out-of-bag prediction from a random forest-based (Breiman, 2001; Svetnik et al., 2003; Knight et al., 2009) QSAR using data on the dopamine D2 receptor downloaded from http://www.bindingdb.org/. In this case we used a random forest learner that was based on the “ensemble tree learner” KNIME node and the full DES encodings, and compared it with the other encodings. The DES encoding was of comparable utility to the other encodings used, although we note that these are log-log plots and that the slope of the lines are rather less than unity, so there would be inaccuracy in linear plots (Kell et al., 2011, 2013; Kell and Oliver, 2014). Figure 9B shows the same QSAR, using only the fractions of the DES encodings indicated. Clearly one can learn very effectively using just the commonest 20% of substructures. Figures 9C,D show a similar analysis for factor Xa inhibition (Fontaine et al., 2005) using data downloaded from http://www.cheminformatics.org/datasets/, while Figure 9E split the data (as did the original authors) into training (out of bag predictions) and test sets as is arguably preferable (Broadhurst and Kell, 2006; Kell and Oliver, 2014). Lastly here (Figure 9F), those data were also split into two output classes based on whether the molecule was a “good” or “poor” inhibitor for factor Xa; obviously the DES encoding admits a highly accurate classifier.

**Figure 10:**
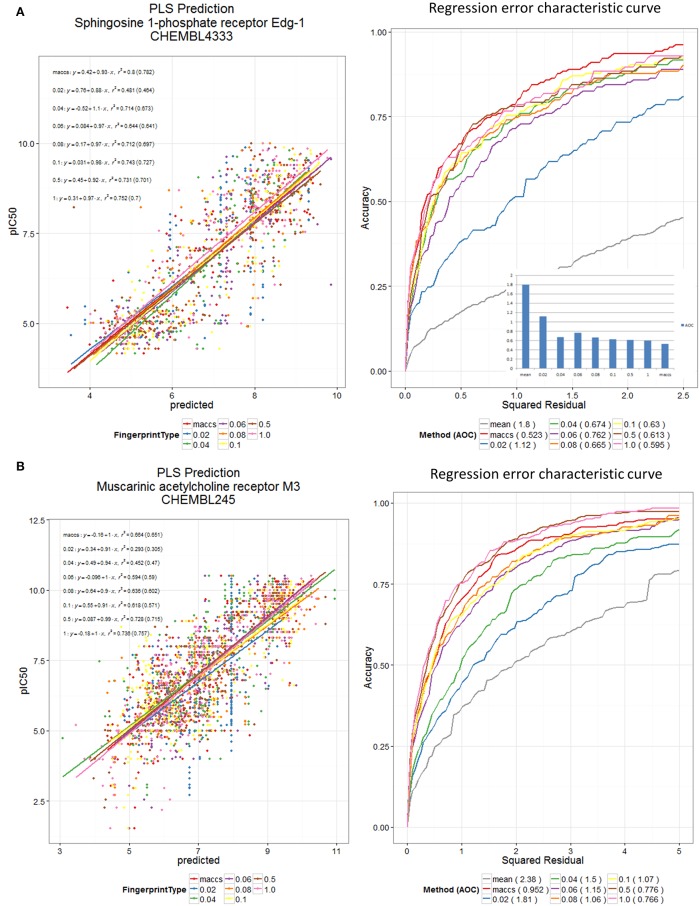
**DES and MACCS encodings predict receptor binding in ChEMBL datasets**. PLS Prediction of **(A)** the ChEMBL 4333 and **(B)** the ChEMBL 245 dataset pIC50 values using DES Fingerprints (for various fractions, N, of scaffolds used) and MACCS Fingerprints. Scaffolds were sorted according to maximum of their (frequency of occurrence in Drugs, frequency of occurrence in Recon2 metabolites). Datasets were split 60:40 into training and test sets, and training data were pre-processed using a low variance filter, and a correlation filter prior to PLS (5 latent variables). The test data were used for plotting the scatter plot and the REC curve. PLS was carried out using the R plsdepot package using the Knime R Integration and Scripting Nodes. The REC curve plot also shows the curve for using the mean value as predictor; this is taken as a reference worst-case method.

Finally, to show the generality of the utility of the new encodings (Figure 10), we used the various encodings to devise quantitative structure-activity relationships for two datasets from the ChEMBL bioactivity database (Bento et al., 2014), here using partial least squares (Wold et al., 2001) and the regression error characteristic (Bi and Bennett, 2003; Mittas and Angelis, 2010) to indicate that reasonable predictions could be obtained by methods other than random forests.

## Conclusions

The concept of drug-endogenite likenesses continues to appear to have utility, and substructure analyses of drugs and endogenites (for which we provide all the data) show both similarities and differences that have led us to implement here a simple substructure-based cheminformatics encoding family, DES, that has a clear and interpretable basis. We note a strong tendency for the Tanimoto similarity metric to favor bitstrings (and hence encodings that lead to them) that are highly populated with ones, and this will bear further analysis. However, we anticipate that variants of the DES encoding may provide useful filters for assessing drug- and endogenite-likenesses and for other cheminformatics purposes.

## Author contributions

DBK and SO'H conceived of the study, participated in its design and coordination and helped to draft the manuscript. SO'H wrote the workflows. All authors read and approved the final manuscript.

## Authors' information

DBK is a Research Professor at the University of Manchester, a role to which he returned full time following a 0.8FTE 5-year secondment at Chief Executive of the Biotechnology and Biological Sciences Research Council. He was previously Director of the Manchester Centre for Integrative Systems Biology (www.mcisb.org). His interests include systems biology, chemical biology, pharmaceutical drug transporters, synthetic biology, and iron metabolism. His website is http://dbkgroup.org and he tweets as @dbkell. At Google Scholar his work has been cited more than 30,000 times, with an H-index of 90. SO'H has a Ph.D. in Chemistry from Warwick University, and following a period in industry is now a Computer Officer at the University of Manchester, specializing in cheminformatics, chemometrics, machine learning and the closed-loop automation of scientific instrumentation.

### Conflict of interest statement

The authors declare that the research was conducted in the absence of any commercial or financial relationships that could be construed as a potential conflict of interest.

## Additional data files

The following additional data are available with the online version of this paper. Additional data file 1 (VolcanoPlotData.xlsx) lists (in order of abundance) all of the substructures extracted from the endogenites and marketed drugs used herein, for which a truncated version is visualized as Figure 7. Additional datafile 2 (Scaffold2DES-Fingerprint.7z)—KNIME node elements for computing the DES encoding(s).
